# The missing link: covalent linkages in structural models

**DOI:** 10.1107/S2059798321003934

**Published:** 2021-05-19

**Authors:** Robert A. Nicholls, Marcin Wojdyr, Robbie P. Joosten, Lucrezia Catapano, Fei Long, Marcus Fischer, Paul Emsley, Garib N. Murshudov

**Affiliations:** a Structural Studies, MRC Laboratory of Molecular Biology, Francis Crick Avenue, Cambridge CB2 0QH, United Kingdom; b Global Phasing Limited, Sheraton House, Castle Park, Cambridge CB3 0AX, United Kingdom; c Netherlands Cancer Institute, Plesmanlaan 121, 1066 CX Amsterdam, The Netherlands; d Oncode Institute, The Netherlands; e Randall Centre for Cell and Molecular Biophysics, Faculty of Life Sciences and Medicine, King’s College London, London SE1 9RT, United Kingdom; f Chemical Biology and Therapeutics and Structural Biology, St Jude Children’s Research Hospital, 262 Danny Thomas Place, Memphis, TN 38105-3678, USA

**Keywords:** covalent linkage, *AceDRG*, CCP4 Monomer Library, restraint dictionary, SARS-CoV-2

## Abstract

Covalent linkages between constituent blocks of macromolecules and ligands have been subject to inconsistent treatment during the model-building, refinement and deposition process. This may stem from a number of sources, including difficulties with initially detecting the covalent linkage, identifying the correct chemistry, obtaining an appropriate restraint dictionary and ensuring its correct application. The analysis presented herein assesses the extent of problems involving covalent linkages in the Protein Data Bank (PDB). Not only will this facilitate the remediation of existing models, but also, more importantly, it will inform and thus improve the quality of future linkages. By considering linkages of known type in the CCP4 Monomer Library (CCP4-ML), failure to model a covalent linkage is identified to result in inaccurate (systematically longer) interatomic distances. Scanning the PDB for proximal atom pairs that do not have a corresponding type in the CCP4-ML reveals a large number of commonly occurring types of unannotated potential linkages; in general, these may or may not be covalently linked. Manual consideration of the most commonly occurring cases identifies a number of genuine classes of covalent linkages. The recent expansion of the CCP4-ML is discussed, which has involved the addition of over 16 000 and the replacement of over 11 000 component dictionaries using *AceDRG*. As part of this effort, the CCP4-ML has also been extended using *AceDRG* link dictionaries for the aforementioned linkage types identified in this analysis. This will facilitate the identification of such linkage types in future modelling efforts, whilst concurrently easing the process involved in their application. The need for a universal standard for maintaining link records corresponding to covalent linkages, and references to the associated dictionaries used during modelling and refinement, following deposition to the PDB is emphasized. The importance of correctly modelling covalent linkages is demonstrated using a case study, which involves the covalent linkage of an inhibitor to the main protease in various viral species, including SARS-CoV-2. This example demonstrates the importance of properly modelling covalent linkages using a comprehensive restraint dictionary, as opposed to just using a single interatomic distance restraint or failing to model the covalent linkage at all.

## Abstract 2

Analysis of the Protein Data Bank revealed that over a third of entries contain covalent linkages without descriptions in the CCP4 Monomer Library (CCP4-ML). The CCP4-ML was updated with *AceDRG* dictionaries corresponding to commonly occurring classes of missing linkages.

## 1. Introduction   

Crystal structures of protein–ligand complexes are often used to infer the chemical basis of biological processes and inform structure-based drug-lead discovery. Hence, it is important to build accurate, reliable models of ligands that give confidence in the interpretation of the respective complex and its interactions. Whilst recent years have seen improvements in tools for ligand description, fitting, analysis and validation (see Nicholls, 2017 ▸ and references therein), difficulties with the treatment of covalently linked compounds have largely remained unaddressed. Consequently, there are a large number of models in the Protein Data Bank (PDB; Burley *et al.*, 2019 ▸) in which the descriptions of covalent linkages are either suboptimal or absent.

Intra-compound stereochemical restraints are automatically handled for common monomers; these are distributed by CCP4 (Winn *et al.*, 2011 ▸) as part of the CCP4 Monomer Library (CCP4-ML), which is also referred to as the *REFMAC*5 (Murshudov *et al.*, 2011 ▸) Dictionary (Vagin *et al.*, 2004 ▸). The CCP4-ML contains entries for standard and non­standard amino acids, nucleotides, saccharides and a large number of compounds that might be present in macromolecular complexes (see Section 3). Other suites, for example those from Global Phasing (Bricogne *et al.*, 2017 ▸) and *Phenix* (Liebschner *et al.*, 2019 ▸), use their own analogous but different libraries, some of which share a partial common ancestry but have diverged over time.

Other novel or less common compounds that do not have a pre-computed description in the CCP4-ML require a custom dictionary to be generated; when using the *CCP*4 suite, the recommended approach is to use *AceDRG* (Long *et al.*, 2017 ▸). *AceDRG* uses a detailed description of local chemistry, along with a modern and extensible underlying data source (Gražulis *et al.*, 2012 ▸). Since the release of *AceDRG*, there has been a need to update the CCP4-ML in accordance with these developments; this is addressed in Section 3.1.

Note that other dictionary-generation software tools are also available, including *eLBOW* (Moriarty *et al.*, 2009 ▸) from *Phenix*, *grade* (Smart *et al.*, 2011 ▸) from Global Phasing and *pyrogen* from *Coot* (Debreczeni & Emsley, 2012 ▸), each of which optionally uses *Mogul* (Bruno *et al.*, 2004 ▸), which utilizes data derived from the Cambridge Structural Database (Groom *et al.*, 2016 ▸); some of these are supplemented with semi-empirical quantum-mechanics (QM)-based approaches. For a comparative summary of these and other similar tools, see Steiner & Tucker (2017 ▸). Note also that restraints for metal ions, notably tetrahedral zinc, can be created automatically by *PDB-REDO* (Touw *et al.*, 2016 ▸) and, where necessary, manual construction of restraint dictionaries for metals can be facilitated by reviewing the data-mined co­ordination preferences and geometric distributions from *MetalPDB* (Putignano *et al.*, 2018 ▸).

Many of the most common inter-monomer covalent linkages have pre-computed descriptions available in the CCP4-ML. These include polymeric linkages (between peptides, nucleotides and saccharides), as well as a number of known common post-translational modifications (for example N- and O-linked glycosylation, disulfide bridges and common metal ion binding); see Appendix *A* [App appa]. There has been a need to add dictionaries corresponding to more covalent linkages to the CCP4-ML. However, since manual consideration is required in order to ensure that linkage descriptions correspond to appropriate chemistries, it has been deemed prudent to prioritize the addition of descriptions for linkage types that most commonly occur in the PDB. Link records (LINK/LINKR/struct_conn; see Nicholls *et al.*, 2021 ▸) are essential during the model-building and refinement process (for non­polymeric linkages). However, the deposition of link records is not considered to be mandatory. Worse, they are currently automatically removed and recalculated by the wwPDB annotation pipeline during deposition, which can cause inconsistencies between model annotation and the modelling assumptions made during structure determination. Ultimately, the model builder is responsible for ensuring the presence and usage of such important annotations. If the automatically generated linkage annotation is incorrect after deposition then the depositor needs to request for the annotation to be revised. Consequently, there is a diverse range of qualities and consistencies with which covalent linkages have been treated in models deposited in the PDB.

In Section 2 we use *Gemmi* (Wojdyr, 2017 ▸) to identify the most commonly occurring covalent linkages found in atomic models in the PDB for which there was no corresponding entry in the CCP4-ML. These include linkages that were assigned during the annotation process (*i.e.* the models contain relevant link records), as well as those that were not explicitly annotated as covalent linkages. Note that since the authors’ original link records are automatically discarded upon deposition, we are unable to reliably distinguish *post hoc* between linkages that were modelled and those that were not modelled as being covalently linked during the structure-determination process. As a result of the analysis presented in Section 2, we have added new linkage descriptions for some of the most commonly occurring missing linkage types to the CCP4-ML. In Section 3 we discuss this recent expansion, as well as the addition of many new, and the replacement of existing, dictionaries using *AceDRG*. In particular, extending the CCP4-ML to include descriptions for commonly occurring linkages (Section 3.3) will facilitate the identification of such linkage types in future modelling efforts, whilst concurrently easing the process involved in their application. In Section 4 the importance of correctly modelling covalent linkages is demonstrated using a case study, which considers an example of current scientific and social importance (the covalent linkage of an inhibitor to the main protease in various viral species, including SARS-CoV-2).

We shall refer here to a ‘model’ as meaning a structural atomic model, unless otherwise stated.

## 2. Analysis of covalent linkages in macromolecular models   

The initial objective of the analysis presented herein is to assess the extent of problems involving covalent linkages in the PDB. Not only will this facilitate the remediation of existing models, but also, more importantly, it will inform and thus improve the quality of future linkages. As a direct consequence of this study, the number of linkage descriptions in the CCP4-ML will be increased. In order to maximize impact, focus will be given to the inclusion of descriptions for the most commonly occurring linkage types.

Firstly, we analyse the covalent linkages that are explicitly annotated in PDB entries, cross-referencing the standard linkage descriptions distributed as part of the CCP4-ML. We then identify linkages that are potentially missing from the PDB; *i.e.* those proximal atom pairs that may be covalently bound, but are not annotated as such. Analysis of such cases aids the prioritization of which linkage descriptions to add to the CCP4-ML. The analysis presented herein was facilitated by *Gemmi* (Wojdyr, 2017 ▸).

### 2.1. Contact analysis and finding potential linkages   

The *Gemmi* library for structural biology, which is primarily designed for working with macromolecular models and crystallographic reflection data, provides tools to facilitate contact analysis. Identification of neighbouring (spatially proximal) atoms involves efficient neighbour searching (Levinthal, 1966 ▸), whilst handling issues relating to (non-)crystallographic symmetry and alternative atomic positions. If the distance between such neighbouring atoms is no greater than the sum of their elemental radii then they are deemed to be in contact; the elemental radius is calculated as a linear function of the covalent radius (Cordero *et al.*, 2008 ▸), thus allowing the detection threshold to both vary with elemental type and be controlled via scaling parameters. Implementation allows filtering based on properties such as whether the contacting atoms belong to the same residue, chain or asymmetric unit, as well as the ability to ignore low-occupancy atoms, H atoms and water molecules.

*Gemmi* has recently been extended to facilitate the identification of potential linkages of known and/or unknown type. This involves using contact analysis to exhaustively identify proximal non-H atom pairs before categorizing them according to whether they correspond to known linkage types by cross-referencing against a set of linkage descriptions (which may be found in the CCP4-ML or in a custom restraint dictionary).

As a general tool for molecular model manipulation, *Gemmi* can also be used to generate and apply link records (LINK/LINKR for PDB format, struct_conn for PDBx/mmCIF format), including those between symmetry-related atoms. Indeed, *Gemmi* can add link records to a model for all linkages of known type that are found using the link-finding algorithm (or otherwise). In addition to their use in the analyses presented in the subsequent subsections, these functionalities of *Gemmi* have also been utilized within graphical interfaces to facilitate the practical application of covalent linkages (Nicholls *et al.*, 2021 ▸).

### 2.2. Analysis of linkages in the PDB   

All models in the PDB derived from X-ray diffraction experiments were analysed using *Gemmi* in order to identify all atom pairs that might potentially be linked, and these were cross-referenced against any annotated link records present in the input models. An atom pair is identified as potentially linked if the interatomic distance *d* is less than a quantity proportional to the sum of the covalent radii of the two atoms (*r*_1_ and *r*_2_; as reported in the CCP4-ML),
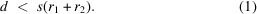
In our implementation, potential linkage identification was performed using an empirically determined scaling factor of *s* = 1.5; the dependency of our results on the chosen value of this parameter should be noted.

When searching for linkages of known type, the standard peptide and phosphodiester bonds (CCP4-ML linkage identifiers ‘TRANS’, ‘PTRANS’, ‘NMTRANS’, ‘CIS’, ‘PCIS’, ‘NMCIS’ and ‘p’) were excluded as these are handled automatically by *REFMAC*5 and thus do not require explicit link records.

When searching for unannotated potential linkages, in order to avoid false-positive hits arising from proximal atom pairs that are covalently bound or separated by only a few covalent bonds, those connected by fewer than four bonds in the chemical graph were excluded. Indeed, if provided with restraint dictionaries for all components and linkages, *Gemmi* can report the graph geodesic distance (the number of edges in the shortest path) between atoms in the chemical graph of a macromolecule. For example, this allowed the exclusion of proximal atoms coordinated by metal ions (for example, the SG atoms in the zinc-binding cysteines C97 and C145 in PDB entry 2oc8; Prongay *et al.*, 2007 ▸); our algorithm avoids the detection of such noncovalently linked atom pairs as being potentially linked.

Since the input models were derived from the PDB, intra-monomer connectivity was inferred from component dictionaries from the wwPDB Chemical Component Dictionary (CCD; Westbrook *et al.*, 2015 ▸). In contrast, inter-monomer connectivity was inferred from link dictionaries from the CCP4-ML. In addition, any atom pairs with an annotated link record were treated as chemically bound when constructing the macromolecular chemical graph. In order to avoid false positives arising from the detection of proximal atom pairs between components that were already covalently bound, atom pairs were excluded from unannotated linkage detection if any other atoms between those two components were already connected (via a connectivity/link record or a CCP4-ML dictionary entry). Furthermore, potential linkages involving residues that contain any atoms with partial (subunitary) occupancies were excluded from the detection of potential unannotated linkages. This allowed the avoidance of a number of pathologies, including overlaid symmetry-related copies of the same atom (for example PDB entry 2vtu; Plevka *et al.*, 2008 ▸) and models of potentially unreliable local quality.^1^

This procedure resulted in four classes of potential linkages: annotated linkages of known type, annotated linkages of unknown type, unannotated linkages of known type and unannotated linkages of unknown type. Here, ‘annotated/unannotated linkage’ refers to the presence of a corresponding link record in the model and ‘known/unknown type’ refers to the presence of a matching link dictionary in the CCP4-ML. We shall herein refer to the resulting identified proximal atom pairs as ‘potential linkages’; such potential linkages may or may not in reality be covalently bound. A summary of the number of identified potential linkages in each category is provided in Table 1 ▸. We find that between around a third (34.2%) and a half (48.3%) of the models in the PDB may contain covalent linkages for which there is presently no corresponding description in the CCP4-ML. Whilst 41.6% of models contain link records, 33.0% of models contain proximal atom pairs that were detected as potentially missing linkages by our algorithm. Whilst the majority of these ‘potentially missing linkages’ may have been false positives, subsequent filtering and analysis allowed the identification of a number of genuine types of covalent linkages (see Section 2.4).

### 2.3. Linkages of known type   

**Figure 1:**
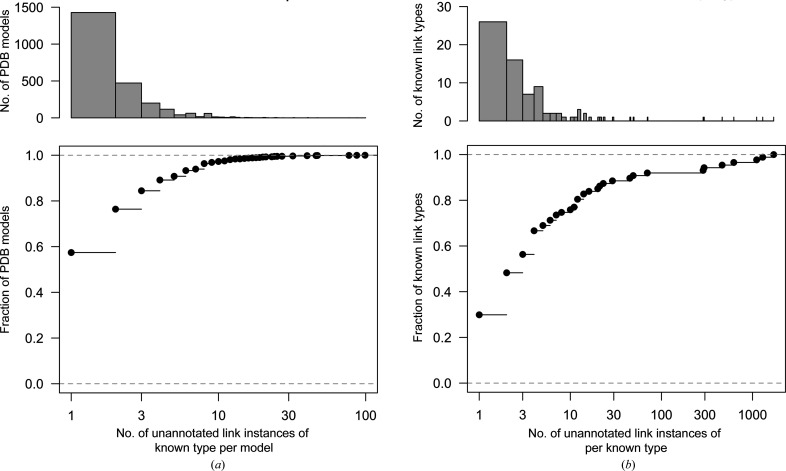
Distributions of the number of unannotated potential linkages of known CCP4-ML type per PDB model (*a*) and per linkage type (*b*). Frequency histograms are shown (top) along with corresponding empirical cumulative distribution functions (bottom). The ten most frequently unannotated known linkage types, the right-most observations in (*b*), are summarized in Table 2 ▸. The horizontal axes are shown on a logarithmic scale. This figure was created using *R* (R Core Team, 2020 ▸).

First, we consider atom pairs identified as potential linkages of known type. Table 1 ▸ indicates that the vast majority of the known link instances (380 687 out of 386 984) in the PDB are annotated using a link/connectivity record. The remaining 6297 unannotated cases are attributed to 2487 PDB entries. It should be emphasized that some of these potentially missing linkages may not in reality be covalently bound; some may be false positives. As seen in Fig. 1 ▸(*a*), over half of these models have only one potentially missing link, whereas some PDB entries have a very large number of missing records (up to 99 in PDB entry 2h6o; in this case all missing linkages correspond to glycosidic bonds and N-linked glycosylation; Szakonyi *et al.*, 2006 ▸).

The identified unannotated potential linkages comprise a total of 87 types of atom pairs for which there are linkage descriptions available in the CCP4-ML. These pertain to 43 unique linkage descriptions; some linkage descriptions can be used for multiple atom pairs (for example, the dictionary for the β-1,2 glycosidic bond, link identifier ‘BETA1-2’, can be found modelling 793 instances of MAN[O2]—NAG[C1], 262 instances of GAL[O2]—FUC[C1] and 80 instances of BMA[O2]—XYP[C1], amongst others). Fig. 1 ▸(*b*) shows that only a single potential link instance appears unannotated for a third of these 43 linkage types; around a half have at most two instances. These might include rare linkage types involving rare components, as well as atom pairs that are modelled as being proximal despite not being truly covalently bonded. However, there are a number of known linkage types for which there are a large number of unannotated link instances in the PDB models. The ten most commonly occurring potentially missing linkage types are exhibited in Table 2 ▸. The vast majority of such missing link records are attributed to disulfide bridges, zinc ion binding, haem C thioether linkages, N-linked glycosylation, glycosidic linkages and iron ion binding.

**Figure 2:**
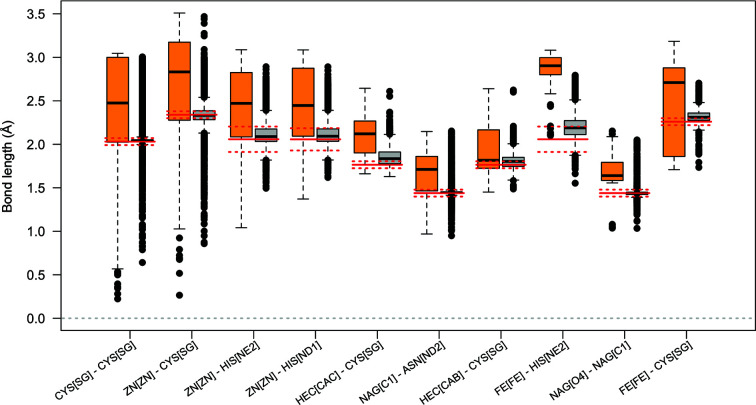
Boxplots representing interatomic distance (bond-length) distributions for the ten most common types of linkage that are unannotated using link/connectivity records in the PDB for which linkage descriptions are available in the CCP4-ML. Linkage types are encoded as ‘Monomer1[Atom1]–Monomer2[Atom2]’; these correspond to the rows in Table 2 ▸. For each linkage type, boxplots are shown corresponding to unannotated (orange, left) and annotated (grey, right) potential linkages. Ideal bond lengths according to the CCP4-ML are shown as solid red lines, with dotted red lines representing two estimated standard deviations from the target values. This figure was created using *R* (R Core Team, 2020 ▸).

The interatomic distance (bond-length) distributions for these ten linkage types are shown in Fig. 2 ▸, highlighting the differences between the distributions for annotated and unannotated linkages. The annotated linkage distributions are generally tight and in agreement with the dictionary values. Linkages involving haem C and iron ions form an exception; one explanation for this may be that the model was refined without an explicit linkage, but a link record was automatically added during deposition. The median values for unannotated potential linkages are significantly longer in all cases. This might be expected, since unrestrained atom pairs are subject to repulsive forces during refinement; here, we assert that there is a strong correlation between the modelling and refinement of covalent linkages and their subsequent annotation by the PDB, although this cannot be verified. The presence of unannotated linkages exhibiting bond lengths close to their optimal values may be an artefact of the wwPDB linkage-annotation protocol changing over time. Note that the short upper tails and the lack of longer outliers in many of the unannotated linkage distributions may be attributed to the choice of proximity-detection threshold. Whether annotated or unannotated, it is evident that there are a number of bond-distance outliers; this reflects the varying quality of models in the PDB. In particular, the abundance of unrealistic disulfide and zinc–cysteine distances is concerning;^2^ the prevalence of such issues has previously been acknowledged (Touw *et al.*, 2016 ▸).

### 2.4. Linkages of unknown type   

We now consider atom pairs identified as potential linkages of unknown type. The identified ‘potential linkages’ may or may not actually correspond to atom pairs that can form covalent bonds. In the case of annotated linkages, the linkage type was deemed to be a valid linkage type by the PDB annotation pipeline, despite not having a corresponding entry present in the CCP4-ML. Information regarding the exact nature, source and treatment of the linkage is not available from the deposited model. Where such potential linkages are unannotated, it is possible that the atom pair should have been modelled and annotated as being covalently linked. Alternatively, it could be that the two atoms are not covalently linked, but rather have drifted together during refinement, ultimately becoming close enough to be identified as a potential linkage by the link-finding algorithm in *Gemmi*.

Whether annotated or unannotated, the covalent linkages may or may not have been modelled and refined as such during structure determination. Restraints for a linkage of unknown type could have been defined in a custom dictionary, or otherwise just a simple link record could have been used in the absence of an appropriate dictionary (which is not a recommended treatment).

When analysing unannotated linkages, it became apparent that a large number of the proximal atoms detected as potentially linked by our algorithm were actually not covalently bonded, and rather were a consequence of the prevalence of inaccurate models in the PDB. In order to reduce the number of such false positives, when analysing linkages of unknown type we considered only the 137 852 PDB entries for which there exists a corresponding pre-computed PDB-REDO entry (version 7.32; van Beusekom *et al.*, 2018 ▸). Consequently, we only further consider entries for which the corresponding data were deposited and the model was able to be successfully automatically re-refined using *REFMAC*5. Since models of poor quality and/or derived using only low-resolution data tend to have high atomic positional uncertainty, those with nominal resolution lower than 3 Å or an *R* factor greater than 30% were excluded (the *R* factor was used as *R*_free_ was not available in too many cases). This resulted in a total of 129 043 PDB model entries being included in our analysis.

In cases where more than one unannotated potential linkage of unknown type was detected between the same two components, only the atom pair with the smallest inter­atomic distance was included. This substantially reduced the number of false positives, although it may have resulted in increased false negatives due to cases where there are multiple linkages between the same two components.

Since high *B* factors compared with other atoms in the structural environment tend to indicate inflated atomic positional uncertainties (Masmaliyeva *et al.*, 2020 ▸), atom pairs were excluded if the *B* factors were much larger than those in the rest in the model (larger than three median absolute deviations from the median; an approximate nonparametric equivalent to two standard deviations from the mean). We also excluded atom pairs if one of the constituents was a water molecule (HOH) or an unknown component (amino acid, UNK; nucleic acid, N; ligand, UNL; atom/ion, UNX). In addition, since one of the primary objectives driving this investigation was to guide expansion of the CCP4-ML using *AceDRG*-derived link dictionaries, and *AceDRG* cannot currently generate dictionaries for metal-containing compounds, metals were excluded from the analysis of un­annotated linkages of unknown type, as were H atoms and nonmetals with atomic number greater than 16. Specifically, potential linkages were only included if both constituent atoms had an element listed as one of B, C, F, N, O, P, S.

Whilst *PDB-REDO* was used to facilitate model-based and atom-based filtering, we used the coordinates corresponding to the original deposited PDB models when searching for potential linkages using *Gemmi*. Therefore, our results pertain to the status of models in the PDB and not the status of those remediated by *PDB-REDO*. A summary of the potential linkages remaining following filtering is shown in Table 3 ▸.

It is evident that a large proportion of the models deposited in the PDB (38.3%) contain link records for which there is no corresponding description in the CCP4-ML. Filtering by *B* factor resulted in a dramatic reduction in the number of identified potential linkages (a 45.3% decrease), especially for linkages that were not annotated (a 63.2% decrease); this is unsurprising due to the tendency of unreliable regions (with high *B* factors) to result in clashing atoms. Excluding potential linkages involving metals, water molecules and unknown components resulted in final set of 11 188 atom pairs being identified as potentially missing linkages, which were found amongst 5739 PDB entries.

Whilst the majority of the identified unknown potential linkage types were found annotated using a link record (4043 types), many of these types were found unannotated (3008 types). A number of these potential types (238) were found both annotated and unannotated amongst the PDB entries.

**Figure 3:**
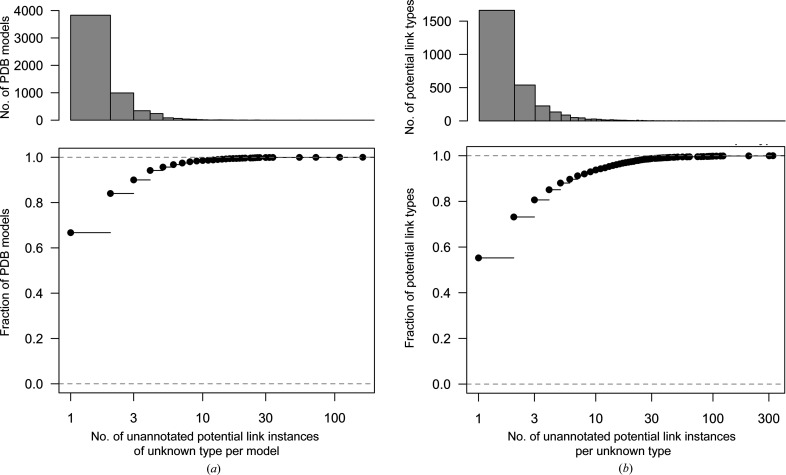
Summary of potentially missing linkages for which there is no corresponding linkage description available in the CCP4-ML. Frequency histograms (top) and the corresponding empirical cumulative distribution functions (bottom) are shown for (*a*) the number of potentially missing linkages per PDB entry (a total of 5739 entries) and (*b*) the total number of individual instances per potential linkage type (a total of 11 188 link instances). The horizontal axes are shown on a logarithmic scale. This figure was created using *R* (R Core Team, 2020 ▸).

The frequency distributions corresponding to the number of PDB model entries and the number of individual link instances for unannotated potential linkages are shown in Fig. 3 ▸. Around two thirds of the 5379 PDB entries only exhibit one such potential missing link; relatively few have more than a few missing linkages. Similarly, over half of the unknown potential linkage types (*i.e.* identified proximal atom-pair types) are only found once amongst all model entries. There are comparatively few potential linkage types for which many individual instances are observed.

**Figure 4:**
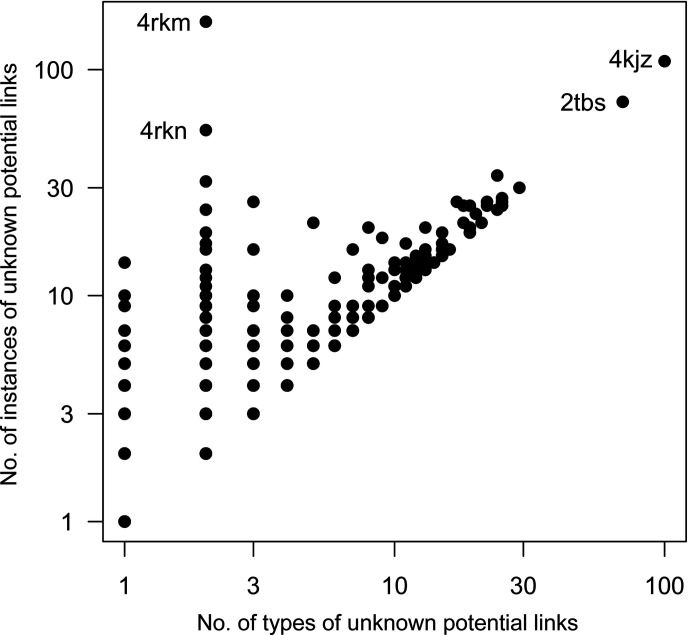
Relationship between the number of potentially missing link instances and the number of unique types of such potential linkages for which there is no corresponding linkage description available in the CCP4-ML. The axes are shown on a logarithmic scale. A total of 5739 models from the PDB were included; the vast majority of these contained only a few link instances (see Fig. 3 ▸). PDB codes are shown for the four models that exhibited the largest frequency of identified potentially missing link instances. This figure was created using *R* (R Core Team, 2020 ▸).

The four PDB entries that exhibited the largest number of potential missing link instances can be seen as the rightmost observations in Fig. 3 ▸(*a*). These are individually identified in Fig. 4 ▸, which illustrates the relationship between the number of potential missing linkage types and the number of individual instances of such missing linkages in a given PDB entry. Whilst the majority of these PDB entries exhibit only one or a few missing linkage types and instances of them, there are a number of entries for which there are a large number of instances of a few types (for example, PDB entries 4rkm and 4rkn) and for which there are a large number of different types (for example, PDB entries 4kjz and 2tbs).

The missing linkages in PDB entries 4rkm (163 instances) and 4rkn (54 instances) correspond to the covalent linkage of cysteine and haem C (Schmitz *et al.*, 2013 ▸). The haem molecules are modelled using the component identifier HEM, which corresponds to haem B. However, in this case the haem molecules are covalently linked to the protein and thus should actually be modelled using the component identifier HEC and annotated using link records to specify the covalent linkage of the HEC[CAB] and HEC[CAC] atoms to the respective CYS[SG] atoms. Since the appropriate HEC–CYS linkages are available as part of the CCP4-ML (see Appendix *A* [App appa]), the appropriate link-restraint dictionaries would have been used during model refinement had the appropriate component identifiers been used and link-connectivity records specified.

PDB entries exhibiting a large number of potential linkage types relative to the number of link instances (the right portion of Fig. 4 ▸) had a tendency to exhibit pathologies that resulted in false positives. In the most extreme case, PDB entry 4kjz (Eiler *et al.*, 2013 ▸), 109 link instances were detected amongst 100 potential linkage types. This model contains a large number of atomic clashes, a result of widespread modelling errors. In another case, PDB entry 2tbs (Smalås *et al.*, 1994 ▸), 72 instances were detected amongst 70 potential linkage types. Again these were false positives; this was a result of clashing symmetry-related molecules that were modelled using unitary atomic occupancies.

When considering which new linkage descriptions to add to the CCP4-ML, further careful manual consideration of the ranked hits, *i.e.* the most commonly occurring potential linkage types, as shown in Fig. 3 ▸(*b*), facilitated the identification of true classes of covalent linkages. Iterative exclusion of PDB entries that were found to contain many false-positive hits (as indicated in Fig. 4 ▸) allowed a further reduction of noise. Using this strategy allowed the avoidance of many false positives, whilst simultaneously maximizing practical impact by focusing on the most common classes of missing linkages. The new linkage descriptions generated as a direct result of this analysis are summarized in Section 3.3.

### 2.5. Linkages involving metals   

Whilst the primary focus of the present investigation is on non-metal-involving linkages, it should be noted that our analysis of models in the PDB also revealed the prevalence of metal-involving linkages (see Table 4 ▸). Of the 575 566 instances in which a link record was used to annotate atomic connectivity, 543 815 can be attributed to linkages involving metals; these were found amongst 33% of PDB model entries.

Whilst the majority of metal-involving linkages are annotated using link records, around 11% are not (42 150 of 369 678 after the exclusion of atoms with high *B* factors). Of the 11 934 PDB model entries that contain unannotated metal-involving potential linkages, the vast majority (10 694) were found to also contain other annotated metal-involving linkages. This reflects the inconsistent treatment/quality of metals within macromolecular models.

Comparison of the bottom rows in Tables 3 ▸ and 4 ▸ indicates that ignoring linkages involving water molecules, the majority of linkages involve metals. This is true for both annotated (29.6% versus 2.5%) and unannotated potential linkages (4.6% versus 1.5%). This highlights the need for future efforts to focus on easing the modelling of metal-involving linkages.

## 3. Modernization of the CCP4 Monomer Library using *AceDRG* dictionaries   

Due to the nature of its historical evolution, having derived from a number of sources as well as being subject to manual curation, the CCP4-ML can be considered to be a heterogeneous source of prior information, which has both expanded and improved since its conception. In this section, we summarize recent efforts to update and consolidate this resource.

In the original release of the CCP4-ML (Vagin *et al.*, 2004 ▸), geometric restraints corresponding to standard polymer-forming monomers were taken from a number of published ‘gold-standard’ reference sources (Engh & Huber, 1991 ▸; Taylor & Kennard, 1982 ▸; Saenger, 1984 ▸; Parkinson *et al.*, 1996 ▸). The program *LibCheck* was created and used to generate restraint dictionaries for commonly occurring non­polymeric monomers (*e.g.* ligands) that were present in the CCD (Westbrook *et al.*, 2015 ▸), resulting in around 2000 component dictionaries. Over time, additional entries corresponding to other monomers were added, mostly using *LibCheck* for dictionary generation. When concerns were raised regarding the quality of individual entries, dictionaries were amended by manual curation.

As of late 2017 (*i.e.* prior to the recent updates described here) the CCP4-ML contained over 13 000 components, 73 linkages and 63 modifications. Many of these linkages and modifications involve the use of component wildcards; these may be applied to the linkage of any components that match the specified component type and atom nomenclature (in the absence of an overriding specialization).

For example, the link description with identifier ‘NAG-SER’ can be applied to any pyranose[C1]–SER[OG] glycosidic linkage (see Appendix *A* [App appa]); the leading component can be any with type listed as ‘pyranose’ irrespective of the monomer identifier code (*i.e.* not just NAG). The PDB contains examples of 14 such matching atom-pair types (with the most common being 289[C1]–SER[OG]). However, there are also dictionary entries for MAN[C1]–SER[OG] and XYS[C1]–SER[OG] (link identifiers ‘MAN-SER’ and ‘XYS-SER’, respectively), which would take precedence over the more general pyranose[C1]–SER[OG] linkage description.

In such cases, where multiple dictionaries are available for a given residue pair, the one that is most specialized should be selected (subject to heuristic geometric configuration matching where relevant, for example *trans*/*cis* for peptide and α/β for glycosidic linkages). More recently, substantial efforts have gone into updating and extending the CCP4-ML in line with developments in *AceDRG* for the creation of component and link dictionaries (Long *et al.*, 2017 ▸; Nicholls *et al.*, 2021 ▸).

### 3.1. Automatically adding/updating nonpolymeric component entries   

*AceDRG* has been used to generate dictionaries for all non-metal-containing nonpolymeric components that are present in the CCD, and the resultant dictionaries have been added to the CCP4-ML. Polymeric components are not included in the automated *AceDRG* updates; they have been considered separately (see Section 3.2). This means that the majority of ligands that were previously modelled in entries deposited in the PDB should now have corresponding entries in the CCP4-ML, thus easing the process involved in their future modelling and refinement.

In cases where the CCP4-ML already contained a given component entry, the corresponding dictionary has been replaced with the new *AceDRG*-generated dictionary where possible. These updated dictionaries should benefit from more appropriate and accurate restraints, due to

(i) the more detailed description of local atomic environments used by *AceDRG* and
(ii) the utilization of a continually expanding wealth of high-resolution structural information in deriving geometric restraints: hundreds of thousands of small-molecule crystal structures.

Such a detailed description of local geometric environments would not be possible were it not for the wealth of underlying prior information. This represents a substantial advancement compared with the approach used in the original derivation of the CCP4-ML component dictionaries.

### 3.2. Manually updated component entries   

Despite the level of automation achieved with *AceDRG*, the maintenance of the CCP4-ML sometimes requires manual intervention. For instance, when new compounds with metals are added to the CCD, restraint files are constructed with *LibCheck* and manually curated. Another task performed manually is the revision of existing geometric targets in the light of new research, with an example being iron–sulfur clusters (Moriarty & Adams, 2019 ▸).

During the course of the present study, we observed many new linkages that could be mapped to existing descriptions (see Appendix *A* [App appa]). This procedure requires explicit typing of compounds, which involves a number of steps.

(i) Checking the topology of the compound to see whether it matches one of the existing types, for example peptide.
(ii) Checking the consistency of atomic nomenclature with other compounds in the category, for example a peptide residue should minimally contain the backbone atoms N, CA, C, O and OXT.
(iii) If the topology and nomenclature of the compound are consistent with a specific category, then the appropriate type can be assigned (peptide, DNA, RNA, pyranose *etc.*).

Since many of the identified unknown linkages involve modified amino acids or nucleotides, the manual typing of compounds quickly resolves such linkages. Amino-acid compounds have been checked manually to ensure consistency of nomenclature; similar work is under way for nucleotides. Detected nomenclature inconsistencies within the CCD were communicated to wwPDB annotators.

### 3.3. Addition of new linkage entries   

Identified potential linkage types were ranked according to their frequency of occurrence, as described in Section 2.4. The most common classes of potential linkages were considered manually in order to ascertain the suitability of modelling particular atom pairs as being covalently bound. Where chemistries were convincing, link dictionaries were generated using *AceDRG*. These new linkages, which are to be added to the CCP4-ML, are summarized in Table 5 ▸. Undoubtedly, a more extensive investigation of the commonly occurring linkage types would produce further additional linkage descriptions.

### 3.4. Inclusion of hydrogen proton positions   

Stereochemical information involving H atoms in the CCP4-ML has been derived from small-molecule X-ray crystallography experiments (acknowledging that some H atoms may have been added in riding positions rather than refined). Consequently, the H—*X* bond-length values (where *X* indicates a non-H atom) reflect the relative positions of electron clouds. It is known that the distances between the nuclei of H and parent atoms are longer than those between the centres of the electron clouds (Coppens, 1997 ▸). In contrast to X-ray macromolecular crystallography (MX), in neutron macromolecular crystallography (NMX) it is the nuclei that scatter. Indeed, in order to facilitate proper modelling of NMX and electron scattering experiments, there has been a need to include geometric information regarding atomic nuclei in component dictionaries.

Thanks to the availability of small-molecule structures solved by NMX^3^ in which the proton positions of H atoms are clearly located, along with the development of more sophisticated calculation methods, it is now possible to determine accurate positions of H nuclei and thus estimate the associated bond lengths. Consequently, in addition to the *AceDRG*-based updates described herein, the component dictionaries in the CCP4-ML are in the process of being extended to include additional information regarding H atoms derived from NMX diffraction data (Allen & Bruno, 2010 ▸) and QM calculations.

Specifically, future CCP4-ML component dictionaries will include two centres for H atoms representing electron and proton positions. This complementary information will be utilized by *REFMAC*5 during the refinement of models against data from NMX, electron diffraction and electron cryo-microscopy reconstructions. In addition, this information will facilitate the analysis and design of nonbonding inter­actions, aiding investigation of the longstanding problem of identifying weak hydrogen bonding. The addition of geometric information regarding hydrogen nuclei to the CCP4-ML will be further detailed elsewhere.

## 4. Case study: covalent linkage between the viral main protease M^pro^ and inhibitor N3   

Structure-based drug-lead design efforts have resulted in a mechanism-based irreversible inhibitor N3 (Yang *et al.*, 2005 ▸), which has been observed to inhibit the main protease (M^pro^) in various coronavirus species, including SARS-CoV (Xue *et al.*, 2007 ▸; Zhang *et al.*, 2010 ▸), MERS-CoV (Ren *et al.*, 2013 ▸), HCoV-HKU1 (Zhao *et al.*, 2008 ▸) and HCoV-NL63 (Wang, Chen, Tan *et al.*, 2016 ▸), as well as in infectious bronchitis virus (Xue *et al.*, 2008 ▸), feline infectious peritonitis virus (Wang, Chen, Liu *et al.*, 2016 ▸), porcine epidemic diarrhoea virus (Wang *et al.*, 2017 ▸) and mouse hepatitis virus (Cui *et al.*, 2019 ▸). Due to its exemplified antiviral properties and as it has been found to be a potent inhibitor of SARS-CoV-2 M^pro^ (Jin *et al.*, 2020 ▸), it is one of the current leads for drug-design efforts attempting to combat the global coronavirus disease (COVID-19) pandemic.

**Figure 5:**
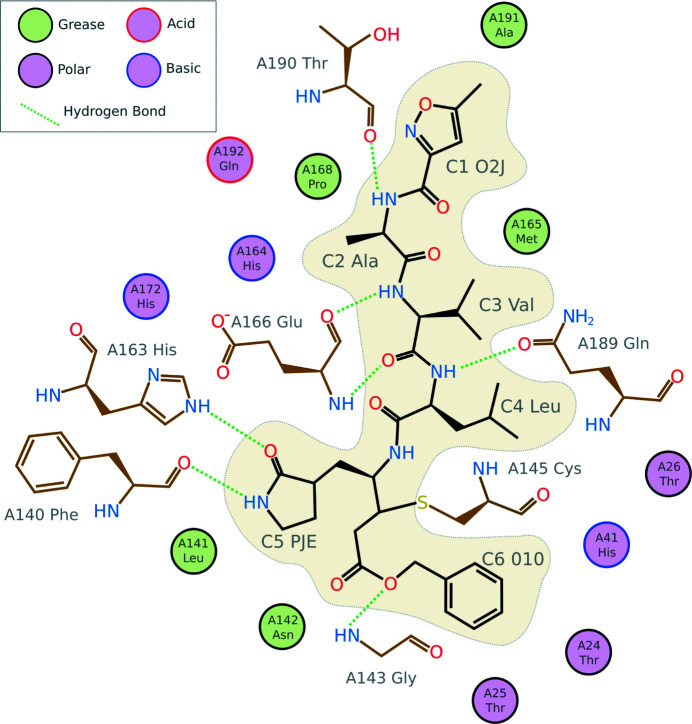
Interaction analysis of N3 binding to SARS-CoV-2 M^Pro^ (PDB entry 6lu7; Jin *et al.*, 2020 ▸) created using *Inkscape* (https://inkscape.org/) based on a template generated using *LigPlot* (Laskowski & Swindells, 2011 ▸).

A thioether bridge (∼1.8 Å) is fundamental in the design of Michael acceptor thiol protease inhibitors (Hanzlik & Thompson, 1984 ▸). In the present context, this relates to the covalent linkage between a cysteine in M^pro^ and carbon C20 in the peptide-like enoic acid compound (component identifier PJE) in the M^pro^ inhibitor N3. This thioether bridge plays an important role in stabilizing the protein–ligand interaction; all other attractive interactions between M^pro^ and N3 involve hydrogen bonds and hydrophobic contacts (see Fig. 5 ▸). Indeed, correct modelling of the covalent linkage is necessary in order to allow accurate inferences about the irreversible nature of the inhibition.

Not only does mismodelling/omitting a covalent linkage affect the interpretation of the interaction itself, it also affects the modelling of other atoms in the surrounding structural environmental network, in terms of both atomic positional and displacement parameters (ADPs; *B* factors; see Section 4.4). In this case, the double bond between atoms C20 and C21 in PJE is changed to a single bond as part of the enzymatic reaction, increasing the internal flexibility of the N3 inhibitor. The correct modelling of the position of a compound and its interactions based on an MX structural model can guide ligand discovery and design. However, incorrect modelling can misinform such campaigns.

### 4.1. Comparative analysis of annotated and unannotated linkages   

Analysis of the 23 proximal^4^ instances of the relevant CYS[SG]–PJE[C20] interaction which were found amongst 11 entries in the PDB revealed that only around half (PDB entries 2hob, 5eu8, 5gwy, 5gwz, 7bqy and 6lu7) of the structures contained explicit link records, whilst in the other five this bond was incorrectly unannotated (PDB entries 2amq, 2q6f, 3d23, 3iwm and 6jij).

**Figure 6:**
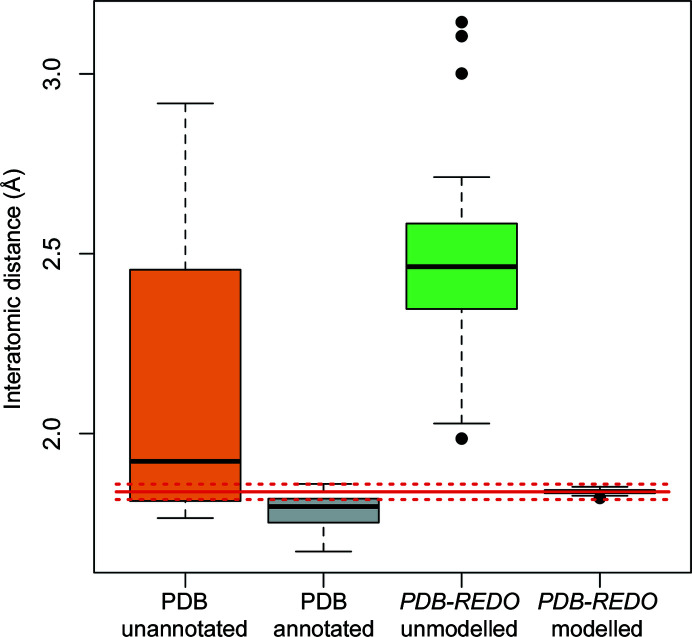
Boxplots representing interatomic distance (bond-length) distributions for the 23 proximal instances of CYS[SG]–PJE[C20] which were found amongst 11 entries in the PDB. Boxplots are shown corresponding to whether the covalent linkage was unannotated (orange; 15 instances in five entries) or annotated using a link record (grey; eight instances in six entries), and also all instances after re-refinement by *REFMAC*5 via *PDB-REDO* without modelling the covalent linkage (green; 23 instances) and modelling the covalent linkage using an *AceDRG* link dictionary (black; 23 instances). The ideal bond length according to *AceDRG* (1.838 Å) is depicted as a solid red line, with dotted red lines representing two standard deviations from the target value (1.817 and 1.859 Å). This figure was created using *R* (R Core Team, 2020 ▸).

The interatomic distance distributions corresponding to these annotated and unannotated covalent linkages are shown in Fig. 6 ▸. The models in which the thioether bridge was unannotated generally exhibited longer distances between the two atoms. It seems reasonable to assert that a lack of annotation correlates with the linkage not being modelled during structure determination (although this cannot be verified). Indeed, a lack of appropriate restraints typically results in atoms becoming separated during model refinement due to repulsive nonbonding forces, as seen when re-refining the models without modelling the covalent linkage (see Fig. 6 ▸). However, some of the unannotated linkages have interatomic distances close to the dictionary value; in such cases it is probable that restraints representing the covalent bond were used during refinement, despite the link record not being present in the PDB model. The exact reasons for the discrepancies between deposited models with link records and the *PDB-REDO* models re-refined using *AceDRG* dictionaries cannot be confirmed, as such information is not available.

### 4.2. Re-refinement using an *AceDRG* link dictionary   

**Figure 7:**
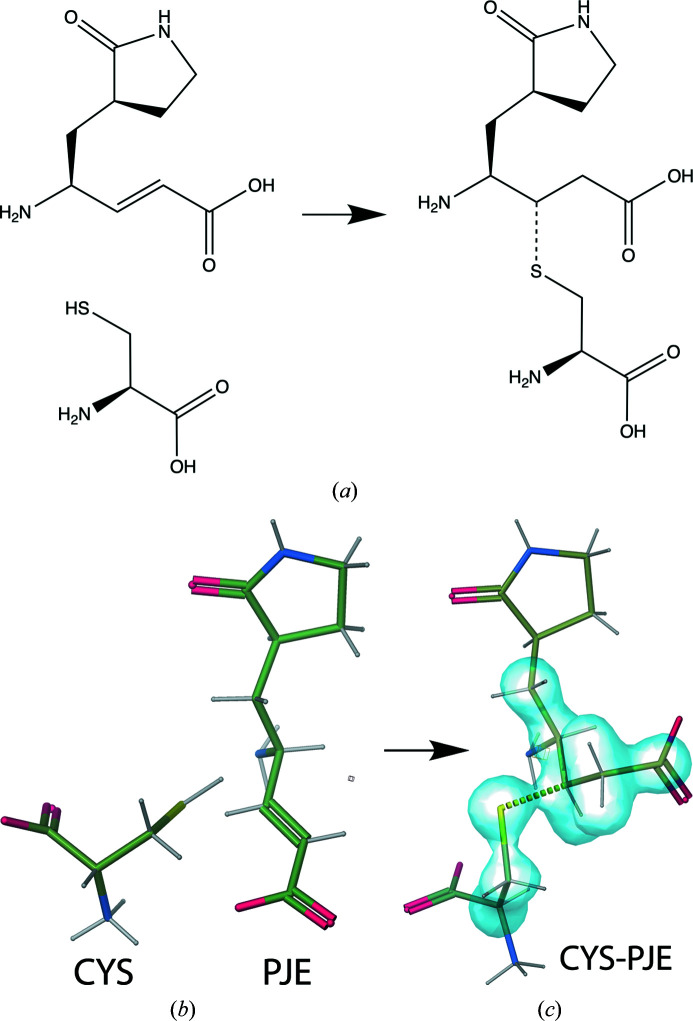
Modelling the covalent thioether linkage between N3 and M^Pro^ using *AceDRG*. (*a*) Diagram of the enzymatic reaction involved in the linkage of CYS[SG] to PJE[C20] (created using *ChemDraw Professional* 17.1). (*b*) Idealized conformers corresponding to the monomers CYS and PJE, as generated using *AceDRG* and distributed in the CCP4-ML. (*c*) The *AceDRG*-generated idealized conformer for the composite component CYS-PJE (figure created using *Coot*; Emsley *et al.*, 2010 ▸). The linkage between the CYS[SG] and PJE[C20] atoms is depicted as a dotted line. The blue transparent surface surrounding atoms in the linked complex highlights which atoms are involved in link dictionary restraints (bond, angle, torsion, chiral and planar restraints); this encompasses both the addition of inter-component restraints as well as modifications applied to the individual components as a consequence of the covalent linkage.

*AceDRG* was used to generate a restraint dictionary for the CYS[SG]–PJE[C20] covalent linkage (see Fig. 7 ▸), as well as for three polymeric linkages within the synthetic N3 inhibitor that are not present in the CCP4-ML (PJE[N5]–LEU[C], PJE[C22]–010[O] and ALA[N]–02J[C41]). Four linkage descriptions have accordingly been added to the CCP4-ML (see Table 5 ▸). After re-refinement using *REFMAC*5 via *PDB-REDO* using the new *AceDRG* link dictionaries, all 23 of the thioether linkage distances were within two standard deviations of the *AceDRG* dictionary target value (see Fig. 6 ▸). The annotated covalent linkages have mean bond lengths of 1.784 Å (σ = 0.0591, *n* = 8), which is close to the ‘ideal’ distance between sulfur and *sp* ^2^ C atoms. Note that this is lower than the *AceDRG* dictionary value (target of 1.838 Å, σ = 0.0107), which is based on an *sp* ^3^ C atom.

**Figure 8:**
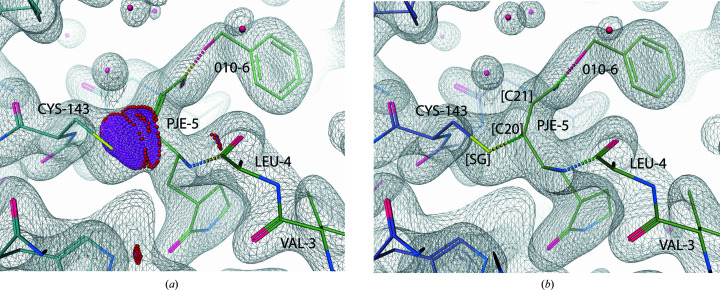
Comparison of the thioether bridge in a PDB annotated model (protein chain *A* and N3 inhibitor chain *D* in PDB entry 2q6f; Xue *et al.*, 2008 ▸) and the same model after re-refinement with *REFMAC*5 using a link record and an *AceDRG* dictionary (figure created using *Coot*; Emsley *et al.*, 2010 ▸). (*a*) The relevant link record was not present in the annotated model; this is reflected in the purple/red *Coot* dots that represent outlier clashes between atoms involved in the thioether bridge; this representation is similar to that produced via *PROBE* (Word *et al.*, 1999 ▸) all-atom contact analysis in *MolProbity* (Chen *et al.*, 2010 ▸). (*b*) After re-refinement using an *AceDRG* dictionary, there are no clashes. Visible residues in the N3 inhibitor are labelled, as is the interacting CYS-143 residue. Atoms CYS-143[SG] and PJE-5[C20] involved in the covalent linkage (dashed line) are labelled in the re-refined model (*b*); for visual clarity these are omitted from (*a*). The atom PJE-5[C21] is also identified; it should be noted that the C20–C21 bond in PJE changes order from double to single when the covalent linkage is modelled correctly.

As exemplified in Fig. 8 ▸, the absence of a link record corresponding to a covalent linkage can often be diagnosed by considering model-validation metrics. The presence of atomic clashes in the original model (Fig. 8 ▸ *a*) indicates that something is wrong, and is a potential indicator of mismodelling (for example not modelling a genuine covalent linkage). In this case, correctly modelling the covalent linkage (Fig. 8 ▸ *b*) results in sensible geometry, a model that fits the density and no atomic clashes.

### 4.3. The effect of covalent linkages on the surrounding stereochemistry   

**Figure 9:**
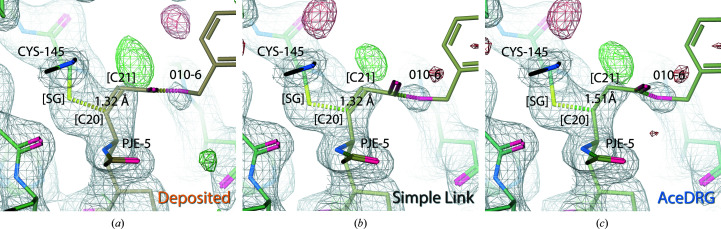
Comparison of the C20–C21 bond in PJE in (*a*) the deposited model (PDB entry 6lu7; Jin *et al.*, 2020 ▸), (*b*) the model re-refined using *REFMAC*5 with a simple link record and (*c*) the model re-refined using *REFMAC5* and an *AceDRG* link dictionary (figure created using *Coot*; Emsley *et al.*, 2010 ▸). In all three cases the C20–C21 bond distance is displayed.

In the standalone PJE monomer there is a double bond between the C20 and C21 atoms (CCP4-ML target value of 1.32 Å, σ = 0.01). However, the C20–C21 bond is changed to a single bond as part of the covalent linkage of CYS[SG] to PJE[C20]. Fig. 9 ▸ considers the most recently deposited model in our test set (PDB entry 6lu7; Jin *et al.*, 2020 ▸), which corresponds to SARS-CoV-2 M^pro^. In the deposited model, the covalent linkage is annotated using a link record. In both the deposited model (Fig. 9 ▸ *a*) and when using a link record but without providing a link dictionary (Fig. 9 ▸ *b*), C20–C21 is modelled as a double bond; this is reflected in refinement to an interatomic distance of 1.32 Å in both cases. Re-refining the model using an *AceDRG* dictionary (Fig. 9 ▸ *c*) results in C20–C21 being modelled as a single bond, resulting in a more realistic interatomic distance of 1.51 Å (*AceDRG* target value of 1.521 Å, σ = 0.011). This emphasizes the point that modelling a covalent linkage not only affects the two atoms involved in the linkage, but that the surrounding chemistry is also affected, highlighting the value of using comprehensive restraint dictionaries when modelling covalent link­ages.

It should be noted that the quality of this particular example case, PDB entry 6lu7, has come under scrutiny due to exhibiting certain pathologies (Wlodawer *et al.*, 2020 ▸). As can be seen in Fig. 9 ▸, there is very little support in the density for the terminal residue 010 of the N3 inhibitor. As highlighted in the *BUSTER* wiki (Bricogne *et al.*, 2017 ▸), residue 010 may be (partially) not present in the crystal, perhaps due to cleavage or radiation damage. Curiously, we note that in the current PDB entry 6lu7 (revision 3.1) the *B* factors of all atoms of the PJE residue are set to a fixed value (20.0 Å^2^). Unlike all other atoms in adjacent residues, it seems that the *B* factors of the PJE atoms were not refined prior to deposition.

Models such as this are currently being used in efforts to combat the COVID-19 pandemic (Sachdeva *et al.*, 2020 ▸). Consequently, in order to optimize interpretability and thus ensure sensible downstream conclusions, it is of importance to ensure that important interactions such as covalent linkages between protein and ligand are modelled, refined and annotated correctly.

### 4.4. *B*-factor analysis   

Analysis of atomic *B* factors can facilitate the identification of model errors (Masmaliyeva *et al.*, 2020 ▸). Typically, proximal atoms tend to have similar *B* factors. Large discrepancies between the *B* factors of proximal atoms can indicate pathologies, *i.e.* incorrect modelling or suboptimal refinement. Consequently, we consider how the modelling of the thioether bridge affects comparative tendencies of the linked atomic *B* factors.

**Figure 10:**
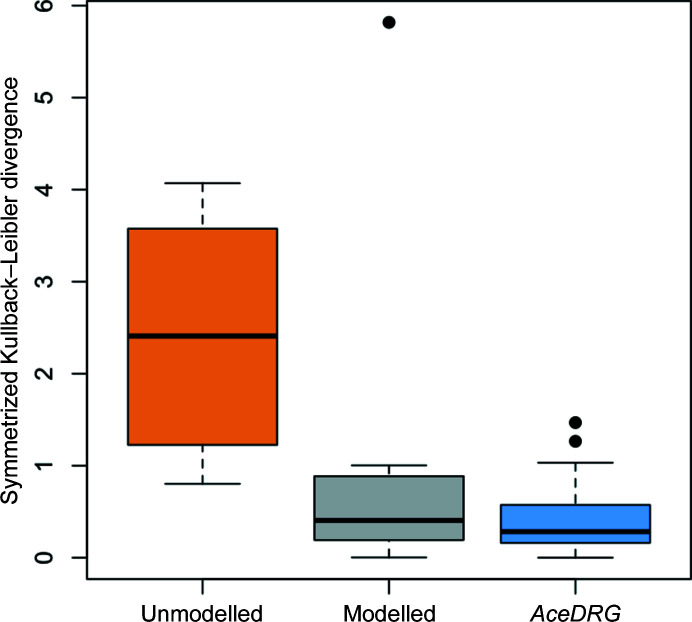
Boxplots representing comparative differences between the *B* factors of linked CYS[SG] and PJE[C20] atoms. All *B* factors were calculated from *PDB-REDO* models. Boxplots show distributions of a symmetrized version of the Kullback–Leibler (sKL) divergence between the *B* factors of the two linked atoms (MacKay, 2003 ▸): if the *B* factors of the SG and C20 atoms are equal then the sKL divergence is zero; higher values indicate greater discrepancies. The three boxplots correspond to linkages that were unmodelled (orange), those that were modelled and annotated using a simple link record but without using a full linkage description dictionary (grey; the outlier corresponds to PDB entry 7bqy; Jin *et al.*, 2020 ▸) and those refined using the new *AceDRG* link dictionary [blue; the outliers correspond to PDB entries 7bqy (Jin *et al.*, 2020 ▸) and 3d23 (Zhao *et al.*, 2008 ▸)]. This figure was created using *R* (R Core Team, 2020 ▸).

In all but one instance of the thioether bridge, the PJE[C20] atom had a larger *B* factor than the CYS[SG] atom. Consequently, it is reasonable to interpret Fig. 10 ▸ as representing the degree to which the *B* factor of the PJE[C20] atom is greater than that of the proximal CYS[SG]; larger values indicate greater relative discrepancies. In order to allow objective comparison, the *B* factors were calculated from *PDB-REDO* models (thus ensuring the similar treatment of *B*-factor parameters during refinement).

It is evident that there are large discrepancies between the *B* factors of atoms involved in unmodelled linkages (orange boxplot in Fig. 10 ▸); the *B* factor of CYS[SG] is typically lower than it should be, as it is forced out of the optimal position by noncovalent repulsive forces (*i.e.* its is still in the basin of attraction corresponding to the SG atom, but is not located exactly at the peak). Concurrently, the *B* factor of PJE[C20] (and other proximal atoms) in the N3 inhibitor will be higher than optimal due to not being appropriately restrained to the M^pro^ via the thioether bridge. This, combined with other incorrect assumptions regarding local chemistry (and of course incorrect atomic positioning) would negatively affect model interpretation.

When the thioether bridge is annotated using a link record, the repulsive (*e.g.* van der Waals) forces are omitted from model refinement. Consequently, the atomic positions refine closer to their optimal values; this is reflected in more consistent *B* factors (grey boxplot in Fig. 10 ▸). However, when the thioether bridge is modelled using a link record and refined using an appropriate restraint dictionary, not only are the repulsive van der Waals forces omitted, but the covalent interaction and its surrounding geometry is also appropriately restrained. As a result, the *B* factors of the interacting atoms become yet again more consistent (blue boxplot in Fig. 10 ▸).

It should be clarified that large *B*-factor inconsistencies are a general indicator of mismodelling, which in our particular case was (largely) a result of not modelling a covalent linkage. In this case, we focused on a particular atom pair (CYS[SG]–PJE[C20]) that we knew was mismodelled to demonstrate how the original authors might have been alerted to such mis­modelling had they looked at the *B*-factor divergence between those proximal atoms. However, it is not necessary to know which particular atoms are covalently bonded. Rather, comparing the *B* factors of any proximal buried nonbonded atoms can lead to the identification of modelling errors.

Also, due to the *B*-factor restraints that are implicitly applied between covalently bonded atoms during refinement (by *REFMAC*5), *B* factors should become more consistent when they are modelled as covalently bonded (compare the orange and grey boxplots in Fig. 10 ▸). However, models refined with an *AceDRG* dictionary generally had lower symmetrized Kullback–Leibler (sKL) divergences^5^ compared with models refined with just a simple link record (compare the grey and blue boxplots in Fig. 10 ▸). Considering that the same *B*-factor restraints were used for both refinements implies that the use of a detailed link dictionary does indeed result in a better model (*i.e.* one with more consistent *B* factors). In other cases, where the assumption of covalent linkage is incorrect, it would be expected that other pathologies indicating mismodelling would become apparent via other forms of local validation.

## 5. Discussion   

Modelling covalent interactions between compounds requires special consideration during macromolecular model building and refinement. It is necessary to have complete chemical knowledge of the system, as well as a corresponding detailed restraint dictionary that describes extended local stereochemistry. Lack of automation and difficulties encountered during modelling have resulted in a large number of the covalent linkages being suboptimally modelled. In addition, failure to model covalent linkages at all has been a prevalent issue. Considering both annotated and unannotated potential linkages, we have investigated the extent of mismodelling of covalent linkages in macromolecular models. We have identified common types of missing linkages and subsequently extended the CCP4-ML with appropriate descriptions.

In order to assess the general extent of problems involving covalent linkages in models in the PDB (Section 2), we have highlighted differences in the distributions of interatomic distances corresponding to covalent linkages of known type (Section 2.3). Whilst the vast majority of such linkages are annotated, there are a substantial number of such linkages that are not annotated using a link record (up to 6297). The vast majority of these can be attributed to only few linkage types; this information may help with prioritizing future efforts towards easing the modelling and validation of common linkages. However, as is indicated by the large number of bond-distance outliers (Fig. 2 ▸), many covalent linkages that have been annotated also exhibit problems. Potential reasons for such pathologies include mismodelling and failure to use a sufficiently comprehensive dictionary to describe the changes to stereochemistry as a result of the chemical reaction; in general, the use of a link record alone is insufficient to achieve a quality model.

Consideration of proximal atom pairs allowed the identification of prevalent classes of potential linkages of unknown type for which there is no corresponding description in the CCP4-ML (Section 2.4). Whilst a number of the identified classes corresponded to false positives, *i.e.* proximal atom pairs that do not form covalent bonds, manual consideration of the most commonly occurring types identified a number of genuine classes of covalent linkages. *AceDRG* link dictionaries were generated for these classes (Table 5 ▸), and corresponding descriptions have been added to the CCP4-ML as a direct consequence of this study. By focusing on the inclusion of new linkage descriptions for the most commonly occurring missing linkages in the PDB, we aim to maximize impact by easing future modelling efforts.

Our analysis also revealed the prevalence of metal-involving linkages in the PDB; these were annotated in 33% and unannotated but potentially present in around 11% of the considered entries (Section 2.5). We found that the majority of linkages in the PDB involve metals. Indeed, there is a need to improve the modelling of metal ions and metal-containing compounds and their interactions with macromolecules. However, there are currently a lack of tools available to facilitate this; the ability to routinely and robustly create restraint dictionaries for metal-containing compounds using *AceDRG* is a future prospect.

The inclusion of new linkage descriptions for identified classes of missing linkages is part of a wider effort to expand and remediate the CCP4-ML (Section 3). Prior to beginning mass updates with *AceDRG* dictionaries in late 2017, there were 13 535 component dictionaries in the CCP4-ML. As of September 2020, 16 618 component dictionaries have been added and 11 179 have been replaced as a consequence of the automated *AceDRG* updates (Section 3.1). There are now over 30 000 component dictionaries in the CCP4-ML: over an order of magnitude more than were distributed in the original release. Only nonpolymeric components are handled as part of the automated updates; other types of components have been manually curated, where possible still using *AceDRG* dictionaries (Section 3.2).

As part of this effort, we observed the atomic nomenclature for modified nucleotides to be less consistent than that for amino acids; there is a need for future remediation efforts to focus on these compounds in the CCD. Indeed, an appropriate compound type can only be assigned if the topology and nomenclature of a compound are consistent with a specific category (for example ‘peptide’, ‘DNA’, ‘RNA’, ‘pyranose’ *etc.*). Where this is not the case, wwPDB annotators can change the CCD compound nomenclature in order to ensure consistency with other entries in the appropriate category. Such a remediation has recently been performed for carbohydrates; this involved the standardization of atomic nomenclature, which in turn allowed us to assign the types ‘pyranose’ and ‘furanose’ to many more compounds (and during the course of the present study we also added a new type, ‘ketopyranose’). This will greatly simplify the future modelling of glycosidic linkages. The prospect of a more general and comprehensive treatment of carbohydrates is on the horizon (Atanasova *et al.*, 2020 ▸). We have focused here on the addition of link dictionaries; the substantial expansion of the CCP4-ML, along with the inclusion of hydrogen proton positions (Section 3.4) and carbohydrate-related improvements, will be further detailed elsewhere.

We have demonstrated the utility of correctly modelling covalent linkages by considering the thioether bridge between an inhibitor N3 and a viral main protease M^pro^ (Section 4). We found the modelling of this linkage to be inconsistent amongst PDB entries (Section 4.1). Using a comprehensive *AceDRG* link dictionary facilitated the correct modelling of the surrounding structural environmental network (Section 4.2). In this case, the covalent linkage affects the modelling of other atoms in the vicinity, notably the bond-order change in one of the linked components (Section 4.3). Finally, we have demonstrated how analysis of the consistency of proximal atomic *B* factors can facilitate the identification of model errors, in this case the mis­modelling of covalent linkages, and have provided additional evidence that the use of comprehensive restraint dictionaries positively affects model quality (Section 4.4).

In the CCP4-ML, we are now attempting to track the provenance of sources of prior knowledge; this is a relatively recent initiative. *AceDRG* component dictionaries populate the pdbx_chem_comp_descriptor mmCIF category, recording which software tools (and versions) were involved in dictionary generation, and thus the source of new and recently replaced CCP4-ML dictionaries can be traced. However, there is still the outstanding issue of how such information propagates to models deposited in the PDB. At present, component and link dictionaries are not deposited, and linkage identifiers that specify the exact dictionary, chemistry and restraints used during refinement are discarded upon deposition. This can have a direct effect on the subsequent interpretability and reproducibility of PDB models. For example, in Section 4.1 we were unable to analyse discrepancies between models owing to a lack of information regarding link-restraint dictionaries for the deposited entries. Hence, one can only speculate about the reasons for any observed discrepancies.

Furthermore, the fact that the original link records of the model authors, and indeed any connectivity annotations, are automatically discarded by the current wwPDB annotation pipeline is a major shortcoming of the deposition process. This meant that we were unable to reliably distinguish *post hoc* between linkages that were and were not modelled as being covalently linked during the structure-determination process. In some cases a deposited model might have been refined without an explicit linkage, but a link record was automatically added during deposition. Conversely, a model might have been refined under the assumption of a covalent linkage, and the link record subsequently discarded during deposition. In addition, since the connectivity annotation may be recalculated as a part of PDB model revision, the persistence of the existing link annotation is not guaranteed. Such inconsistencies between modelling assumptions during the structure-determination process and after deposition can lead to subsequent misinterpretation of the qualitative nature of macromolecular complexes.

The historical need for such a treatment by the wwPDB, *i.e.* the automatic removal of link records, has been due to the heterogeneous qualitative nature of models deposited in the PDB; technical difficulties have been encountered when interpreting the information within submitted PDBx/mmCIF files that originate from different sources. This could be addressed by the adoption of a universal standard for the specification of connectivity records (including identifiers) and associated changes to wwPDB deposition recommendations/policy. Compliance with such standards would allow the connectivity annotation by the authors to be properly encapsulated, and noncompliance could be reported in the wwPDB validation report.

Given that the CCP4-ML is now using *AceDRG* dictionaries, and different software suites may favour other sources of prior information (for example, traditional references such as Engh & Huber, 1991 ▸), we emphasize that there will be qualitative differences between models that were refined using different software. Consequently, we further recommend that the PDBx/mmCIF format be extended in order to allow the encapsulation of restraints used as prior information, or at least a reference to the original sources, during deposition in the PDB. In this context, it should also be noted that there is a need to ensure cross-compatibility between different suites: the typical use of multiple tools from multiple suites should not result in a loss of information regarding provenance. In the *CCP*4 suite, linkage identifiers are currently specified using LINKR records in PDB format and using the ccp4_link_id data item in PDBx/mmCIF format models (Nicholls *et al.*, 2021 ▸). However, looking to the future, we would encourage the adoption of a universal convention for the modelling of covalent linkages.

We emphasize that it is important to properly model covalent linkages using a comprehensive restraint dictionary, as opposed to just using a single interatomic distance restraint, or indeed failure to model the covalent linkage at all. Addressing problems involving covalent linkages will facilitate future modelling efforts and ultimately improve the interpretation of structural data for biology and drug discovery.

## Figures and Tables

**Table 1:** Summary of potential linkages in PDB models: proximal noncontiguous atom pairs Number of identified individual instances of potential linkages, and the number of PDB entries to which they belong, using the *Gemmi*-based algorithm described in Section 2.2. Percentages relative to the total number of PDB entries included in the analysis are shown in parentheses; a total of 166 944 PDB entries were included in the analysis (after excluding 419 due to containing unknown ligands marked with the residue name UNL or being otherwise invalid/unsuitable for processing). Potential linkages are categorized according to whether they are known (linkage type description present in the CCP4-ML) and annotated (link record present in the model). Data pertain to the status of the PDB as of September 2020.

		Annotated	Unannotated	Total
Known	PDB models	46397 (27.8%)	2487 (1.5%)	46853 (28.1%)
	Link instances	380687	6297	386984
Unknown	PDB models	57029 (34.2%)	54132 (32.4%)	80665 (48.3%)
	Link instances	698765	365871	1064636
Total	PDB models	69571 (41.6%)	55027 (33.0%)	
	Link instances	1079452	372168	

**Table 2:** Most commonly unannotated linkage types in the wwPDB Summary of the ten most common types of linkage that are unannotated using link/connectivity records in the PDB for which linkage descriptions are available in the CCP4-ML (the corresponding linkage identifiers are shown as they appear in the CCP4-ML). The linkage types are sorted according to the number of potential linkages identified; these correspond to the rightmost extreme observations in Fig. 1 ▸(*b*). For each linkage type, a representative example of an instance of the given covalent linkage in an existing PDB entry is provided.

Description	Linkage ID	Monomer 1	Atom 1	Monomer 2	Atom 2	No. of cases	Example
Disulfide bridge	Disulf	CYS	SG	CYS	SG	1708	1x3s, A110–A155
Zinc–cysteine link	ZN-CYS	ZN	ZN	CYS	SG	1294	5aoj, B1300–B238
Zinc–histidine link	ZN-HISNE	ZN	ZN	HIS	NE2	1110	4bt5, A301–A194
Zinc–histidine link	ZN-HISND	ZN	ZN	HIS	ND1	623	4q7r, A304–B25
Haem C thioether link	HEC-CYS1	HEC	CAC	CYS	SG	464	2zxy, A200–A15
N-linked glycosylation	NAG-ASN	NAG	C1	ASN	ND2	315	6jgb, A701–A221
Haem C thioether link	HEC-CYS2	HEC	CAB	CYS	SG	287	2zxy, A200–A12
β-1,4 GlcNAc–GlcNAc	BETA1-4	NAG	O4	NAG	C1	96	5lwx, A605–A607
Iron–histidine link	FE-HISNE	FE	FE	HIS	NE2	70	3zku, A1328–A214
Iron–cysteine link	FE-CYS	FE	FE	CYS	SG	45	2pya, A54–A9

**Table 3:** Summary of potential linkages of unknown type in PDB models for entries that are present in the PDB-REDO databank Number of identified individual instances of potential linkages of unknown type, and the number of PDB entries to which they belong, using the *Gemmi*-based algorithm described in Section 2.2. Potential linkages are categorized according to whether they are annotated (link record present in the model). Data pertain to the status of the PDB as of September 2020. A total of 129 043 PDB entries present in the PDB-REDO databank with a nominal resolution no lower than 3 Å and an *R* factor no higher than 30% were included. The corresponding frequencies are also shown following the incremental removal of atoms with high atomic *B* factors and those involving metal/water/unknown components, as described in Section 2.4. Percentages relative to the total number of unknown potential link instances (753 273) and PDB entries included in the analysis (129 043) are shown in parentheses.

		Annotated	Unannotated	Total
Resolution ≤ 3 Å, *R* factor ≤ 30%	PDB models	49467 (38.3%)	43056 (33.4%)	67250 (52.1%)
	Link instances	575566 (76.4%)	177707 (23.6%)	753273 (100.0%)
Atomic *B*-factor filtered	PDB models	37954 (29.4%)	21539 (16.7%)	46129 (35.7%)
	Link instances	346445 (46.0%)	65348 (8.7%)	411793 (54.7%)
Exclude metal/water/unknown	PDB models	6300 (4.9%)	5739 (4.4%)	11575 (9.0%)
	Link instances	18914 (2.5%)	11188 (1.5%)	30102 (4.0%)

**Table 4:** Summary of potential linkages of unknown type involving metals in PDB models for entries that are present in the PDB-REDO databank Number of identified individual instances of potential linkages of unknown type involving metals, and the number of PDB entries to which they belong, using the *Gemmi*-based algorithm described in Section 2.2. Percentages relative to the total number of unknown potential link instances (753 697) and PDB entries included in the analysis (129 043) are shown in parentheses.

		Annotated	Unannotated	Total
Resolution ≤ 3 Å, *R* factor ≤ 30%	PDB models	42627 (33.0%)	20320 (15.7%)	44825 (34.7%)
	Link instances	543815 (72.2%)	95342 (12.7%)	639157 (84.9%)
Atomic *B*-factor filtered	PDB models	33361 (25.9%)	11934 (9.2%)	34601 (26.8%)
	Link instances	327528 (43.5%)	42150 (5.6%)	369678 (49.1%)
Exclude water/unknown	PDB models	31606 (24.5%)	10860 (8.4%)	33004 (25.6%)
	Link instances	223182 (29.6%)	34651 (4.6%)	257833 (34.2%)

**Table 5:** Summary of new linkage descriptions added to the CCP4-ML New linkage descriptions, generated using *AceDRG*, are informally categorized in order to aid assimilation. Formal linkage identifiers are provided, along with the monomer/component and atom identifiers corresponding to the two linked atoms. For each linkage type, the frequencies of identified annotated and unannotated potential linkages are shown and a representative example instance in an existing PDB entry is provided. Where linkages are generic rather than specific to a particular monomer, the component type to which they are applied is provided (the table includes generic linkages involving components classed as ‘peptide’, ‘pyranose’, ‘dna/rna’ and the new type ‘ketopyranose’; these are the types as they appear in the CCP4-ML). The full list of linkage descriptions presently distributed in the CCP4-ML is provided in Appendix *A* [App appa].

Category	Linkage ID	Monomer 1	Atom 1	Monomer 2	Atom 2	Annotated	Unannotated	Example
Lysine linkages	LYS-CYS	LYS	NZ	CYS	SG	0	354	6h27, B73–B70
	LYS-ASN	LYS	NZ	ASN	CG	139	82	4oq1, A264–A354
	LYS-RET	LYS	NZ	RET	C15	383	17	5u6g, G108–G201
	LYS-PLP	LYS	NZ	PLP	C4A	1264	17	3b8w, A34–A1001
	Pept-LYS	peptide	C	LYS	NZ	101	119	5emz, A76–B48
Histidine linkages	HIS_TYR1	HIS	ND1	TYR	CB	61	60	4enu, C392–415
	HIS_TYR2	HIS	NE2	TYR	CE2	68	64	3abk, A240–A244
	HIS-FAD1	HIS	ND1	FAD	C8M	121	15	1w1s, A105–A1535
	HIS-FAD2	HIS	NE2	FAD	C8M	171	19	6b58, A44–A601
Tyrosine linkages	MET-TYR	MET	SD	TYR	CE1	20	29	5whs, A264–A238
	TRP-TYR	TRP	CH2	TYR	CE2	10	17	5whq, A90–A238
Cysteine linkages	CYS-CYC	CYS	SG	CYC	CAC	326	48	4xxi, A196–A301
	CYS-PEB	CYS	SG	PEB	CAA	215	57	3v58, B158–B204
	CYS-FAD	CYS	SG	FAD	C8M	150	23	5zao, A343–A501
Disulfide bridges	Ddisul	DCY	SG	DCY	SG	134	0	5e5t, B29–B62
	CYS-BME	CYS	SG	BME	S2	437	201	3cav, A148–A329
Peptide to peptide-linking	Pept-GYC	peptide	C	GYC	N	75	14	3ls3, B61–B62
	Pept-CR8	peptide	C	CR8	N	115	2	3s05, C61–C64
	Pept-MDO	peptide	C	MDO	N	122	0	3kdy, B151–B152
	Pept-CRO	peptide	C	CRO	N1	314	3	5mak, D64–D66
	Pept-NRQ	peptide	C	NRQ	N1	131	0	4h3l, A65–A66
	Pept-NH2	peptide	C	NH2	N	1097	31	6q5p, F30–F31
Peptide-linking to peptide	GYC-Pept	GYC	C	peptide	N	89	0	6nqj, C63–C65
	CR8-Pept	CR8	C	peptide	N	118	0	4ljd, C64–C65
	MDO-Pept	MDO	C	peptide	N	119	1	6hqf, A205–A203
	CRO-Pept	CRO	C3	peptide	N	315	5	5mak, B66–B68
	NRQ-Pept	NRQ	C3	peptide	N	149	2	3nt3, C63–C66
	IAS-Pept	IAS	CG	peptide	N	126	0	1dy5, A67–A68
Glycosidic and DNA/RNA linkages	ALPHA2-6	ketopyranose	C2	pyranose	O6	132	3	4fqc, H310–H308
	GTP-p	GTP	O3’	dna/rna	P	237	1	4gv9, E8–E9
N3 inhibitor linkages	02J-ALA	02J	C41	ALA	N	23	0	See Section 4
	PJE-010	PJE	C22	010	O	23	0	
	PJE-CYS	PJE	C20	CYS	SG	8	12	
	PJE-LEU	PJE	N5	LEU	C	23	0	

**Table 6:** Summary of pre-existing linkage descriptions in the CCP4-ML Linkages are informally categorized in order to aid assimilation. Formal linkage identifiers are provided, along with the monomer/component and atom identifiers corresponding to the two linked atoms. Where linkages are generic rather than specific to a particular monomer, the component type to which they are applied is provided. The CCP4-ML includes generic linkages involving the following component types: ‘polymer’, ‘peptide’, ‘P-peptide’ (proline-like), ‘M-peptide’ (*N*-methylated), ‘pyranose’ and ‘dna/rna’ (written as they appear in the CCP4-ML). This table corresponds to the present state of the CCP4-ML, prior to inclusion of the new linkage descriptions to be added as a consequence of the work undertaken for the present article. A number of obsolete link descriptions are omitted (‘XYPa-XYP’, ‘XYP-BMA’, ‘BR-C5’, ‘CH3-O2*’ and ‘DM1-CH2’). The new linkage descriptions presently being added to the CCP4-ML are summarized in Table 5 ▸. This list may evolve; for an up-to-date list of linkages in future versions of the *CCP*4 suite, along with their descriptions, refer directly to the CCP4-ML (see the data_link_list section in $CCP4/lib/data/monomers/list/mon_lib_list.cif).

Category	Linkage ID	Monomer 1	Atom 1	Monomer 2	Atom 2
Peptide bond					
Generic C–N linkage	LINK_C-N	polymer	C	polymer	N
	LINK_CNp	polymer	C	peptide	N
	LINK_CpN	peptide	C	polymer	N
*Trans* peptide bond	TRANS	peptide	C	peptide	N
	PTRANS	peptide	C	P-peptide	N
	NMTRANS	peptide	C	M-peptide	N
*Cis* peptide bond	CIS	peptide	C	peptide	N
	PCIS	peptide	C	P-peptide	N
	NMCIS	peptide	C	M-peptide	N
Peptide-like					
To peptide	AHT-ALA	AHT	N2	ALA	CB
	DFO_C-N	DFO	C	peptide	N
	DFO_N-C	peptide	C	DFO	N
	STA_C-N	STA	C	peptide	N
	STA_N-C	peptide	C	STA	N
To peptide-like	STA_DFO	STA	C	DFO	N
	DFO_STA	DFO	C	STA	N
	STA_STA	STA	C	STA	N
	DFO_DFO	DFO	C	DFO	N
	TPN-TPN	TPN	C′	TPN	N1′
To methylamine	DFO-NME	DFO	C	NME	N
	STA-NME	STA	C	NME	N
	NME_N-C	NME	C	peptide	N
Peptide to functional groups					
Formyl	FOR_C-N	FOR	C	peptide	N
	FOR_C-C	peptide	C	FOR	C
	FOR-LYZ	LYZ	NZ	FOR	C
Acetyl	ACE_C-N	ACE	C	peptide	N
Glutamyl	ILG_CD-N	ILG	CD	polymer	N
	ILG_CD-p	ILG	CD	peptide	N
Miscellaneous involving peptides					
Peptide-linking to peptide	ALA-SNN	peptide	C	SNN	N3
	PEP-ORN	peptide	C	ORN	NE
	VAL-SUI	peptide	C	SUI	N
	SUI-GLN	SUI	C	peptide	N
Nonpolymer to peptide	IVA_C-N	IVA	C	peptide	N
	BOC_C-N	BOC	C	peptide	N
	SFN-TYR	SFN	S	TYR	OH
	HEC-CYS1	HEC	CAC	CYS	SG
	HEC-CYS2	HEC	CAB	CYS	SG
Nonpolymer to peptide-linking	ACYSNN	ACY	CH3	SNN	N1
	DG9-SER	DG9	P2A	SER	OG
Disulfide bridges	SS	peptide	SG	peptide	SG
	disulf	CYS	SG	CYS	SG
	MPR-CYS	MPR	SG	CYS	SG
	CYS-MPR	CYS	SG	MPR	SG
Metal ions	ZN-CYS	ZN	ZN	CYS	SG
	ZN-HISND	ZN	ZN	HIS	ND1
	ZN-HISNE	ZN	ZN	HIS	NE2
	FE-CYS	FE	FE	CYS	SG
	FE-HISND	FE	FE	HIS	ND1
	FE-HISNE	FE	FE	HIS	NE2
	SF41-CYS	FE1	FE1	CYS	SG
	SF42-CYS	FE2	FE2	CYS	SG
	SF43-CYS	FE3	FE3	CYS	SG
	SF44-CYS	FE4	FE4	CYS	SG
	SF31-CYS	FE1	FE1	CYS	SG
	SF32-CYS	FE2	FE2	CYS	SG
	SF33-CYS	FE3	FE3	CYS	SG
	SF34-CYS	FE4	FE4	CYS	SG
Carbohydrates					
Glycosidic linkages	ALPHA1-2	pyranose	O2	pyranose	C1
	ALPHA1-3	pyranose	O3	pyranose	C1
	ALPHA2-3	ketopyranose	C2	pyranose	O3
	ALPHA1-4	pyranose	O4	pyranose	C1
	ALPHA1-6	pyranose	O6	pyranose	C1
	BETA1-2	pyranose	O2	pyranose	C1
	BETA1-3	pyranose	O3	pyranose	C1
	BETA2-3	ketopyranose	C2	pyranose	O3
	BETA1-4	pyranose	O4	pyranose	C1
	BETA1-6	pyranose	O6	pyranose	C1
Glycosylation	MAN-SER	MAN	C1	SER	OG
	NAG-SER	pyranose	C1	SER	OG
	FUC-THR	FUC	C1	THR	OG1
	NAG-THR	pyranose	C1	THR	OG1
	MAN-THR	MAN	C1	THR	OG1
	NAG-ASN	pyranose	C1	ASN	ND2
	MAN-ASN	MAN	C1	ASN	ND2
	XYS-THR	XYS	C1	THR	OG1
	XYS-SER	XYS	C1	SER	OG
	XYS-ASN	XYS	C1	ASN	ND2
Nucleic acids and methylation					
Phosphodiester bond	p	dna/rna	O3′	dna/rna	P
Metal ion	MG-O1P	MG	MG	dna/rna	O1P
	MG-O2P	MG	MG	dna/rna	O2P
Methyl/methylene	CH3-N1	CH3	C	dna/rna	N1
	CH2-N2	CH2	CH2	dna/rna	N2

## Footnotes

1 Atom pairs belonging to residues containing any partial occupancy atoms were excluded from the analysis due to concerns regarding the tendency of such residues to be locally unreliable and thus liable to cause excessive pollution with false positives. Manual consideration of several such instances demonstrated the judiciousness of this approach. For example, consider the disulfide bridge between residues B61 and B91 in PDB entry 3hzw (Fernandes *et al.*, 2010 ▸), in which some of the atoms in those two residues are modelled with varying occupancies; it was deemed appropriate to exclude such models that lack realistic physical interpretation from our analysis.

2 Examples include the 0.6 Å disulfide bridge between L23 and L88 and the surrounding stereochemistry in PDB entry 2cju (Scotti & Gherardi, 2006 ▸), and the use of link records to model only two of the four covalent bonds between zinc J101 and the coordinating cysteines in PDB entry 3gtg (Wang *et al.*, 2009 ▸).

3 For example, there are presently over 2300 NMX structures in the CSD.

4 A relaxed interatomic distance threshold of 4.0 Å was used for this analysis. Note that three of the identified covalent linkages (in PDB entry 3iwm) had an interatomic distance greater than 2.7 Å, exceeding the threshold used for automatic detection in the analysis presented in Section 2. Consequently, these three instances do not contribute to the figures presented in Table 5 ▸.

5 The sKL divergence is calculated as 3[(*B*_1_/*B*_2_) + (*B*_2_/*B*_1_) − 2]. This formulation is used by *REFMAC*5 in the design of isotropic *B*-factor restraints. An analogous calculation for anisotropic *B* factors is described by Murshudov *et al.* (2011 ▸).

## Appendix A Pre-existing linkage descriptions

A summary of pre-existing linkage descriptions in the CCP4-ML is given in Table 6 ▸.
